# Clinico-Metabolic Profile in Lean Versus Obese Polycystic Ovarian Syndrome Women

**DOI:** 10.7759/cureus.37809

**Published:** 2023-04-19

**Authors:** Nidhi Makhija, Surekha Tayade, Shikha Toshniwal, Hard Tilva

**Affiliations:** 1 Department of Obstetrics and Gynaecology, Jawaharlal Nehru Medical College, Datta Meghe Institute of Higher Education and Research, Wardha, IND

**Keywords:** menstrual disorders, hirsutism, body mass index, obesity, polycystic ovarian syndrome

## Abstract

Background

Polycystic ovarian syndrome (PCOS), which affects women of reproductive age, is the most prevalent endocrine disorder. Signs of excessive androgen, irregular menses, prolonged anovulation, and infertility are characteristics of the clinical phenotype. Women with PCOS are more likely to have diabetes, obesity, dyslipidemia, hypertension, anxiety, and depression. PCOS affects women's health starting before conception and continuing through their post-menopausal years.

Methods

Ninety-six study subjects were recruited from women visiting the gynaecology clinic according to the Rotterdam criteria for PCOS. Study subjects were then divided into lean and obese groups according to their body mass index (BMI). Demographic data, and obstetrical and gynaecological history were obtained including marital status, menstrual cycle regularity, recent abnormal weight gain (in the preceding six months), and subfertility. To identify any clinical signs of hyperandrogenism such as acne, acanthosis nigricans, or hirsutism, a general and systemic examination was conducted. Data were analyzed after the clinico-metabolic profile was assessed, compared, and contrasted between the two groups.

Results

The findings showed a significant correlation between obese women with PCOS and the clinical profile of PCOS i.e. menstrual irregularities, acne vulgaris, acanthosis nigricans and hirsutism; the waist-hip ratio was higher in both groups. Higher levels of fasting insulin, fasting glucose: insulin ratio, postprandial sugars, homeostasis model assessment of insulin resistance (HOMA-IR) index, total testosterone, free testosterone, and luteinizing hormone/follicle-stimulating hormone (LH: FSH) ratio were seen in obese women with PCOS, whereas the levels of fasting glucose, serum triglycerides, serum high-density lipoprotein cholesterol (HDL) were higher in all the study subjects irrespective of BMI.

Conclusion

The study showed that women with PCOS have a deranged metabolic profile like abnormal blood sugar, insulin resistance (IR), and hyperandrogenemia with clinical derangements like irregular menses, subfertility, and recent weight gain more frequently with higher BMI.

## Introduction

Polycystic ovarian syndrome (PCOS) is a common heterogeneous disorder that affects women and causes irregular menstruation, prolonged anovulation, and the development of hyperandrogenic symptoms. The prevalence ranges from 6% to 20% in women of reproductive age group as described by various studies [1]. The prevalence may vary further depending on the diagnostic criteria and definitions used.

PCOS symptoms typically first appear in the early pubertal years. Anovulation, irregular menstrual cycles, and acne are the defining symptoms. Knowing the triggering reasons is difficult due to complex interwoven pathophysiology. There have been numerous documented mechanisms implicated in the pathophysiology of PCOS. Hypothesized causes of the illness include metabolic abnormalities such as obesity, insulin resistance (IR), and compensatory hyperinsulinemia, and complex interactions between the hypothalamic-pituitary-ovarian or hypothalamic-pituitary-adrenal [2].

A common finding in PCOS is a higher body mass index (BMI), which is primarily linked to IR and elevated insulin levels. Around 40%-60% of PCOS-afflicted females are overweight or obese [2,3]. Moreover, it seems that there is a connection between insulin levels and PCOS severity [4]. According to several research, women with PCOS have higher BMIs [5]. PCOS women are at higher risk for obesity even though it is a global health issue that is becoming more widespread [6]. The increased prevalence in PCOS women of obesity is mostly due to environmental and genetic factors, including high-calorie meals and inactivity [7-9]. Hence, it is challenging to determine whether obesity causes PCOS or vice versa. PCOS is closely linked to metabolic dysfunctions [10] through complex mechanisms. Abnormal plasma glucose values are suggested to be due to IR which is commonly associated with PCOS, as also reported by several studies [11,12]. This is reflected as higher insulin levels and deranged glucose: insulin ratio after the glucose challenge [13]. Understanding the connections between these metabolic dysfunctions and higher BMI would allow for a better appreciation of interventions such as optimizing the weight profile of women with PCOS for effective management. Several researchers have also studied dyslipidemia reported in women with PCOS and attributed the incidence to hyperandrogenism, IR [14,15], and obesity.

Therefore, the current study was conducted to assess women with PCOS and correlate it with clinico-metabolic profile and BMI.

## Materials and methods

The current hospital-based observational study was conducted over a six-month period at the Obstetrics and Gynecology Department of Jawaharlal Nehru Medical College's Acharya Vinoba Bhave Rural Hospital in Sawangi (Meghe), Wardha. Women in the reproductive age range made up the study's population. This study was approved by Datta Meghe Institute of Medical Sciences (Deemed to be University); DMIMS(DU)/IEC/2020-21/9345.

Consenting women between the ages of 15 and 45 with PCOS met the inclusion criteria, according to Rotterdam guidelines [16]. Women who are expecting, those with known endocrine disorders like Cushing's syndrome, 21-hydroxylase deficiency, congenital adrenal hyperplasia, thyroid dysfunction, hyperprolactinemia, and diabetes, as well as those who use oral contraceptives, antiandrogens, glucocorticoids, antihypertensives, anti-diabetics, and anti-obesity medications; smokers; those who chew tobacco; and those who use illicit drugs were excluded from the study.

Study participants were women who visited gynaecology clinics and had menstrual complaints such as irregular menses, delayed menses, hypomenorrhea (reduced menstrual bleeding), weight gain, excessive body hair growth, acne, or a report from an ultra-sonography suggesting polycystic ovaries. Their complete history was taken, clinical examinations and pelvic ultrasonography were done to see if they met the study's eligibility requirements. To diagnose PCOS using Rotterdam criteria, trans-vaginal and/or trans-abdominal ultrasonography was conducted to observe uterine and ovarian morphology, particularly the number and size of follicles [16]. Menstrual cycle history, obstetric history, historical, personal, and family histories, as well as any prior treatments or investigations, were thoroughly recorded. Participants with chronic conditions such as hypertension, diabetes, renal disorders, or malignancies were excluded from the study.

Age, education, occupation, residence location, and socioeconomic level were all recorded as part of the demographic data. The history of obstetrics and gynaecology was gathered, including information on subfertility, recent abnormal weight increase, menstrual cycle regularity, and marital status. To identify any clinical signs of hyperandrogenism, such as acne, acanthosis nigricans, or hirsutism, a general and systemic examination was conducted.

After performing a thorough general physical examination and gynaecological examination, the measurements of height (cm), weight (kg), waist circumference (WC) (cm), and hip circumference were taken. The waist-hip ratio and Ferriman-Gallwey scores were computed.

Body weight (kg)/square height is the formula used to calculate BMI. Lean PCOS is defined as having a BMI of 25 (kg/m2) or below, whereas obese PCOS is defined as having a BMI >=25 (kg/m2) [17].

**Figure 1:**
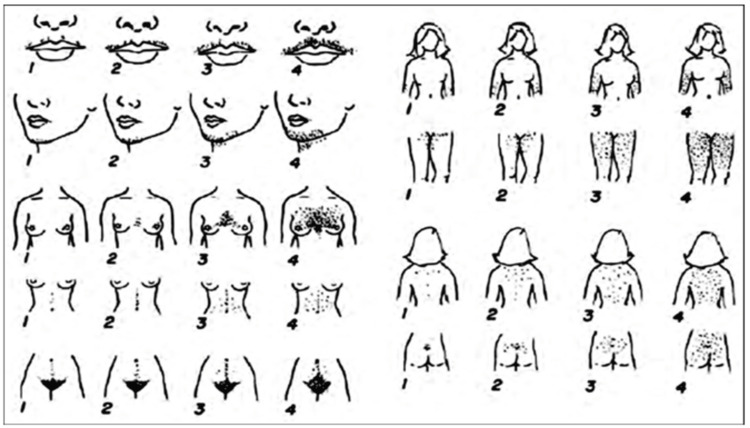
Modified Ferriman-Gallwey score [2]

In 11 locations of the body, the modified Ferriman-Gallwey score was employed to evaluate hair growth (Figure 1). Maximum growth was graded as 4+ and the absence of terminal hair growth as 0. A sum of 8 or greater was deemed to indicate hirsutism.

Biochemical investigations

We assessed the levels of fasting serum insulin (FSI), two hours after a meal, and fasting plasma glucose (FPG). The fasting glucose to insulin ratio was established. Using the aforementioned findings, the homeostasis model assessment of insulin resistance (HOMA-IR), a marker of insulin resistance, was calculated using the formula: HOMA-IR = FPG (mmol/L) x FSI (mIU/mL)/22.5.

Between the third and fifth day of a naturally occurring or progesterone-induced menstrual cycle, morning venous blood samples were drawn between 9 and 10 am (after an overnight fast of 8 to 12 hours). The serum levels of luteinizing hormone (LH), dehydroepiandrosterone sulphate (DHEAS), total testosterone (TT), and free testosterone were measured.

Using the Abbott ArchitectC16000analyzer's photometric assays, high-density lipoprotein cholesterol (HDL) and serum triglycerides (TG) were measured. To measure the concentrations of free testosterone, we used the radioimmunoassay method.

## Results

The demographic profile of the study subjects is shown in Table 1. Maximum study subjects (63.5%) belonged to the age group of 21-30 years, with a mean of 25.4 ± 5.2 years and a range of 16-37 years. Maximum study subjects i.e. 40 (41.46%) were undergraduates and were students by occupation and the majority i.e. 36 (37.5%), belonged to the lower middle class according to the modified Kuppuswamy scale. The mean age of menarche was 13.27 years with a range of 11-15.5 years. Fifty (52.1%) were married, out of which 30 (60%) had subfertility. Maximum, 66 (68.75%) were obese (BMI (≥ 25 kg/m2) and 30 (31.25%) were lean with a mean BMI of 26.3 ± 3.38 kg/m2.

**Table 1:** Demographic profile of study subjects

Parameter	Number	Percentage
AGE (in years)		
< 20 years	25	26.0
21-30 years	61	63.5
31-40 years	10	10.5
MARRITAL STATUS		
Married	50	52.1
Unmarried	46	47.9
LITERACY		
Primary Education	16	16.6%
Secondary Education	24	25.0%
Undergraduate	40	41.6%
Graduate and above	16	16.6%
SOCIOECONOMIC STATUS		
Upper	6	6.25%
Upper Middle	10	10.4%
Lower Middle	36	37.5%
Upper Lower	35	36.4%
Lower	9	9.37%
AGE OF MENARCHE		
≤ 13 years	66	68.75
13.1 - 14 years	18	18.75
>14 years	12	12.12
OCCUPATION		
Students	40	41.6
Homemaker	31	32.2
Unskilled worker	2	2.08
Skilled worker / Professional	23	23.9
BODY MASS INDEX		
Lean (< 25 kg/m2)	30	31.25
Obese(≥ 25 kg/m2)	66	68.75
Mean BMI	26.3 ± 3.38	

Correlation of clinical profile and BMI (lean versus obese) in women with PCOS is shown in Table 2. Menstrual irregularities were frequent in women with PCOS having higher BMI (54 (81.8%) in obese PCOS versus 7 (23.4%) in lean PCOS; p < 0.001). Acne vulgaris was seen in 60 (90.9%) obese women with PCOS versus 17 (56.6%) lean (p=0.0328); Acanthosis nigricans was seen in 19 (28.7%) obese women with PCOS versus one (3.33%) lean (p<0.004); hirsutism was seen in 60 (91%) obese women with PCOS versus 20 (66.7%) lean (p=0.0031). Raised waist-hip ratio (cut off >0.80 [8]) was seen in 70 (72.9%) women with PCOS irrespective of BMI, 51 (77.2%) in obese versus 19 (63.3%) in lean; p=0.1542).

**Table 2:** Correlation of clinical profile and body mass index (lean versus obese) in women with PCOS Lean PCOS = BMI <25 kg/m^2^, obese PCOS = BMI ≥ 25 kg/m^2^, WHR = waist hip ratio, PCOS = polycystic ovarian syndrome. *p<0.05 is statistically significant

Parameter	Lean PCOS (n=30)	Obese PCOS (n=66)	Total (n=96)	p value
Menstrual Irregularity	7 (23.4)	54 (81.8)	61(63.5)	<0.001
Acne Vulgaris	17 (56.6)	60 (90.9)	77 (80.2)	0.032
Recent weight gain	2 (7.7)	34 (51.5)	36 (37.5)	<0.001
Acanthosis Nigricans	1 (3.33)	19 (28.7)	20 (20.8)	0.004
Modified Ferriman Gallwey score ≥8	20 (66.7)	60 (91.0)	80 (83.3)	0.003
WHR >0.80	19 (63.3)	51 (77.2)	70 (72.9)	0.154

Correlation of metabolical profile and BMI (lean versus obese) in women with PCOS is shown in Table 3. In obese women with PCOS, mean fasting insulin was greater than in lean women (15.01 ± 1.53mIU/ml versus 13.9 ± 1.9mIU/ml; p=0.048). When compared to lean women without PCOS, obese women with PCOS had a higher mean fasting glucose: insulin ratio (7.5 ± 1.90 versus 8.2 ± 2.39; p=0.045). In obese women with PCOS, mean post-meal glucose levels were higher than in lean women [144 ± 23.9 versus 155 ± 21.9 mg/dl; p=0.032]. In comparison to lean women without PCOS, obese women with the condition had a higher mean HOMA-IR index (5.45 ± 2.11 versus 2.95 ± 0.76; p<0.001). When compared to lean women without PCOS, obese women with PCOS had higher mean total testosterone levels (169.33 ± 14.8mg/dl versus 136.53 ± 18.65 mg/dl; p<0.001). In obese women with PCOS, mean free testosterone levels were greater than in lean women (4.84 ± 1.29ng/dl versus 3.69 ± 0.49ng/dl; p<0.001). In obese women with PCOS, the mean LH: FSH ratio was greater than in thin women (2.48 ± 0.57 versus 1.12 ± 0.48; p<0.001).

**Table 3:** Correlation of metabolical profile and body mass index (lean versus obese) in women with PCOS Lean PCOS = BMI <25 kg/m^2^, obese PCOS = BMI ≥ 25 kg/m^2^, HOMA-IR = homeostasis model assessment-insulin resistance, HDL-c = high-density lipoprotein cholesterol, LH = luteinizing hormone, FSH = follicle-stimulating hormone, PCOS = polycystic ovarian syndrome. *p<0.05 is statistically significant

Parameter assessed	Lean PCOS (Mean ± SD)	Obese PCOS (Mean ± SD)	p value
Fasting Plasma Glucose (mg/dl)	105.9 ± 17.7	124 ± 13.37	0.534
Insulin Fasting(mIU/ml)	13.9 ± 1.91	15.01± 1.53	0.048
Fasting Glucose: Insulin ratio	7.5± 1.90	8.2± 2.39	0.045
Post prandial glucose (mg/dl)	144 ± 23.9	155 ± 21.9	0.032
HOMA IR	2.95 ± 0.76	5.45 ± 2.11	0.001
Serum Triglyceride (mg/dl)	164 ± 26.24	172 ± 26.67	0.174
Serum HDL-c (mg/dl)	36.33 ± 4.45	34.8 ± 4.84	0.144
Total Testosterone (ng/dL)	136.53 ± 18.65	169.33 ± 14.8	<0.001
Free Testosterone (ng/dL)	3.69 ± 0.49	4.84 ± 1.29	<0.001
LH/FSH ratio	1.12 ± 0.48	2.48 ± 0.57	<0.001

Mean fasting glucose was high in women with PCOS irrespective of BMI [124 ± 13.37mg/dl in obese versus 105.9 ± 17.7mg/dl in lean PCOS (p=0.534)]. Mean serum triglyceride was high in women with PCOS irrespective of BMI (172 ± 26.67mg/dl in obese versus 164 ± 26.24mg/dl in lean; p=0.174). Mean serum HDL was low in women with PCOS irrespective of BMI [34.8 ± 4.84mg/dl in obese versus 36.33 ± 4.45mg/dl in lean; p=0.144).

## Discussion

Almost 10% of women in the reproductive age group suffer from the primary gynecological endocrine illness known as PCOS, which is also steadily increasing [18-21]. Chronic anovulation, symptoms of hyperandrogenism, and characteristics of polycystic ovaries as shown on an ultrasonogram are the major characteristics. Insulin resistance, metabolic syndrome, and obesity are the defining features of this illness.

Several studies suggest that women with PCOS are overweight and obese [22-24]. Women with PCOS are at higher risk for obesity, despite the fact that it is a global health issue that is becoming more widespread [25]. In the present study, out of 96 women with PCOS, the majority, i.e., 41 (42.7%) had a BMI of 25-29.9 kg/m2 (overweight), followed by 25 (26.0%) with a BMI of >30 kg/m2 (obese). More women with PCOS had higher BMI. Most of the studies reported similar BMI levels in the overweight and obesity range, with a mean of 30.1 ± 4.2 Kg/m2 reported by Ali et al. [26] and 31.32 ± 4.80 Kg/m2 by Hamed et al. [27]; they found that BMI was significantly higher in PCOS cases.

There is a strong correlation between PCOS with obesity, especially central obesity, as reported by previously conducted studies [26,27]. Similar results were reported by Abdelazim et al. [28], who found that 35.5% of PCOS women were obese and 44.5% of PCOS women were overweight. The high prevalence of obesity in women with PCOS is largely caused by a combination of environmental and genetic variables, including high-calorie meals and inactivity [29-31]. Hence, it is challenging to determine whether obesity causes PCOS or vice versa.

Few researchers have also studied women with PCOS in the two BMI categories of lean PCOS with obese PCOS with a cut-off BMI of 25 kg/m2. In the present study, 30 (31.25%) study subjects belonged to the lean category of BMI and 66 (68.75%) to the obese category. The mean BMI was 26.3 ± 3.38kg/m2. The findings of the present study are similar to Ali et al. (2021), Hamed et al. (2021), Faeza et al. (2022), and Caltekin et al. (2021) [26,27,32,33].

Acne, hirsutism, and Acanthosis nigricans in PCOS

In the current study, 77 (80.2%) women with PCOS had acne vulgaris, 20 (20.8%) had acanthosis nigricans and 80 (83.3%) had hirsutism. All these clinical parameters were commonly found in women with higher BMI. It is not surprising that acne is one of the main cutaneous manifestations of PCOS since hyperandrogenism is one of the syndrome's main characteristics. According to Aljefri et al. (2021), the most prevalent cutaneous symptoms in 212 (47.3%) and 182 (40.6%) patients, respectively, were hirsutism and acne vulgaris [34]. Also, they discovered that 20% to 40% of acne sufferers also have PCOS. Findings similar to the present study were reported by Gainder et al. (2019) and Chuan et al. (2010) [35,36]. The reportedly higher prevalence of acne, hirsutism, and acanthosis nigricans in PCOS is linked with the endocrinal abnormalities that underlie PCOS. These dermatologic manifestations are a manifestation of hyperandrogenic state in PCOS [23].

Menstrual irregularity

Menstrual irregularities, high BMI and PCOS have been studied by various researchers to establish an association. In the present study, seven out of 30 (23.4%) lean women with PCOS had irregular menstrual pattern as compared to 54 out of 66 (81.8%) obese women with PCOS with a statistically significant difference (p<0.001) showing that menstrual irregularities were frequent in PCOS women having higher BMI. Similar findings have been reported by Aljefri et al. (2021) and Guh et al. (2009) [34,37]. Studies have reported that menstrual irregularity and variability in the length of menstrual cycle is the hallmark of PCOS [28,38]. The irregular pattern of menstrual cycle in obese PCOS is contributed by the fluctuating hormonal levels, especially a lack of optimal LH surge that affects ovulation and the luteal phase. Consequently, the latter half of menstrual cycle gets prolonged, thereby increasing the length of menstrual cycles. This could be an effect of the obese and overweight status of the individuals [34,38].

Metabolic parameters in PCOS 

PCOS is closely linked to metabolic dysfunctions; in the present study, fasting Insulin, fasting sugar: insulin ratio and HOMA-IR were significantly higher in obese women with PCOS (p value <0.0001). An abnormal post prandial glucose in obese PCOS was significantly higher as compared to non-obese ones (p value 0.003). Similar findings have been reported by Nath et al. (2019) and Hamed et al. (2022) [12,27]. Despite the complexity of PCOS, insulin resistance (IR) is a crucial factor in its emergence. During a fast, the pancreas secretes more insulin, which helps to maintain normal blood glucose levels [8]. In response to a glucose challenge, the pancreas secretes more insulin, however, this insulin level might not be sufficient to maintain a normal blood sugar level. After a glucose challenge, this is demonstrated by increased glucose, disordered GTT, and insulin levels [27].

In present study, serum testosterone, free testosterone, and LH:FSH ratio were significantly higher in women with PCOS having higher BMI (p value = 0.041). Hyperandrogenism was also reported with PCOS by Nath et al. (2019) and Hamed et al. (2022) [12,27]. In the study by Hamed et al. [27], serum testosterone levels were also positively correlated with estradiol (r=0.372, p<0.0001) in cases with higher BMI. The values for both estradiol and testosterone were significantly higher in PCOS patients with higher BMI levels. Furthermore, Nath et al. also mentions that raised estrogen levels in obese PCOS are due to peripheral aromatization seen in adipose tissue that directly impacts LH release, thereby increasing FSH:LH ratio [12]. Hamed et al. also revealed alterations in LH and FSH in obese PCOS patients as compared to lean ones [27].These findings are in partial agreement with other studies which also suggested that LH rise is due to higher GnRH pulse frequency [31,32].

Present study also found increased triglyceride levels and reduced high density lipoproteins (HDL-c) in women with PCOS, with a statistically significant difference in lean versus obese PCOS women (p-value 0.17, 0.14 respectively). Moreover, Hamed et al. (2020) demonstrated a substantial increase in TC, LDL-C, and TG blood concentrations and a significant decrease in HDL-C serum values in PCOS patients compared to controls, indicating aberrant lipid profiles in PCOS patients [27]. Up to 70% of PCOS women have dyslipidemia, a common metabolic condition. Dyslipidemia in PCOS women was reported by Kiranmayee et al. (2017) and Javed et al. (2019), who linked the condition to hyperandrogenism and IR [14,15].

## Conclusions

Women with PCOS have irregular menses, subfertility, and recent weight gain, especially when BMI is high and have a high waist-hip ratio regardless of weight. Dermatologic manifestations of acne vulgaris, hirsutism and acanthosis nigricans are seen in women with PCOS, more commonly when BMI is high. Deranged metabolic profile like abnormal blood sugar, insulin resistance, and hyperandrogenemia is seen in PCOS women, especially when BMI is high. Hypertriglyceridemia is seen in PCOS women irrespective of weight profile. Thus, higher BMI leads to metabolic derangements in PCOS women and weight reduction and lifestyle changes are important factors in the management of PCOS.
